# The relationship between vitamin D and chemotherapy-induced toxicity – a pilot study

**DOI:** 10.1038/bjc.2012.194

**Published:** 2012-05-15

**Authors:** D Kitchen, B Hughes, I Gill, M O'Brien, S Rumbles, P Ellis, P Harper, J Stebbing, N Rohatgi

**Affiliations:** 1 Leaders in Oncology Care (LOC), 95 Harley Street, London W1G 6AF, UK; 2 Barts and The London School of Medicine and Dentistry, Garrod Building, Turner Street, London E1 2AD, UK; 3 Guys and St Thomas NHS Foundation Trust, Cancer Management Office, 4th Floor, Bermondsey Wing, Guy's Hospital, Great Maze Pond, London SE1 9RT, UK; 4 Department of Medical Oncology, Imperial College and Imperial College Healthcare NHS Trust, Fulham Palace Road, London W6 8RF, UK

**Keywords:** vitamin D, chemotherapy, toxicity, side effects

## Abstract

**Background:**

There are anecdotal data that lower levels of vitamin D may be associated with increased levels of toxicity in individuals receiving chemotherapy; we therefore wished to investigate this further.

**Methods:**

From a cohort of over 11 000 individuals, we included those who had vitamin D levels (serum 1,25(OH)_2_D3) measured before and during chemotherapy. They were analysed for side effects correlating Chemotherapy Toxicity Criteria with vitamin D levels, normalising data for general markers of patient health including C-reactive protein and albumin.

**Results:**

A total of 241 (2% of the total cohort) individuals entered the toxicity analysis. We found no overall difference in toxicity effects experienced by patients depending on whether they were vitamin D depleted or had sufficient levels (*P*=0.78).

**Conclusion:**

This pilot study suggests routine vitamin D measurement during treatment does not appear to be necessary in the management of chemotherapy-induced toxicity.

Vitamin D deficiency and its relation to chronic illness including cancer has been the subject of much debate. Some argue that vitamin D deficiency is central to the causation of cancer and influential in the pathophysiology at a molecular level (Liu *et al*, 2003; Schwartz *et al*, 2005; Ingraham *et al*, 2008; Manson *et al*, 2011). However, many suggest that the evidence is, at best, inadequate to recommend universal measurement and replacement. Although there has been limited data suggesting that vitamin D deficiency may be a co-factor in chemotherapy-induced mucocutaneous toxicity (Fink, 2011), to the best of our knowledge no studies have been conducted to test the association between vitamin D depletion and chemotherapy toxicity. We therefore conducted a pilot study to examine this association and asked the question ‘Does chemotherapy cause more toxicity amongst patients with vitamin D depletion?’

## Methodology

The oncology cohort at the Leaders in Oncology Care clinic in London has over 11 000 patients with data prospectively recorded for the period between May 2005 and September 2011. When patients attend the clinic for treatment their toxicities are recorded electronically on the MOSAIQ electronic medical records package (Elekta, Stockholm, Sweden) using the Chemotherapy Toxicity Criteria (CTCAE3.0) scale (Trotti *et al*, 2003). Of the cohort, 455 (4%) patients had their vitamin D levels analysed using Diasorin Liaison machines (Saluggia, Italy) for serum 1,25(OH)_2_D3. The reference range of normal values was >75 nmol l^−1^. Values between 25 and 75 nmol l^−1^ were considered insufficient and those <25 nmol l^−1^ considered deficient. The insufficient and deficient groups were combined to form an ‘abnormal’ group.

A total of 241(2%) patients had vitamin D levels measured within 6 months of receiving chemotherapy, excluding biological and hormonal therapies. We studied all ⩾grade 2 chemotherapy-related toxicities among these patients using the CTCAE3.0 scale as our reference. We recorded the most common toxicities seen and those that had the most significant impact on the quality of life. We documented them among various cancer types including palliative, neo-adjuvant and adjuvant chemotherapies.

Statistical analyses to examine differences between the groups were performed using the statistical analysis package SPSS, version 18 (IBM, Armonk, NY, USA). Using Pearson's *χ*^2^ analysis, *P*-values of ⩽0.05 were interpreted as significant.

To avoid bias because of pro-inflammatory state and general poor health, we also recorded the C-reactive protein (CRP) and albumin of these patients and assessed their medians to see if the groups were comparable. These data were also analysed for various cancer types to look for any unusual trend in specific cancers.

## Results

A total of 165 (68%) patients of the overall study group were found to have experienced at least one ⩾grade 2 toxicity effect, 29 of 41 (71%) in the normal group and 136 of 200 (68%) in the abnormal group. The most common overall toxicity effects observed were pain, fatigue and dry skin. Fatigue, hand–foot reactions and dry skin were the most common toxicity effects in the normal group whereas pain, fatigue and hearing loss were the three most frequent toxicities among patients within the abnormal vitamin D group. Also, at least one toxicity effect (rated ⩾grade 2) was recorded among 71% of patients in the normal group, 62% in the insufficient group and 74% in the deficient group.

Upon comparison of the normal and abnormal groups, we were not able to identify any statistically significant difference in the total incidence of toxicities recorded (*P*=0.78). This was also shown on comparison of normal, insufficient and deficient groups (Supplementary Table S1).

Dry skin (17% *vs* 10%), hand–foot reactions (14% *vs* 5%) and mucositis (10% *vs* 6%) had higher percentage frequencies in the normal group compared with the abnormal group. Although conversely, neuropathy (7% *vs* 2%), hypertension (6% *vs* 0%) and thrombosis (8% *vs* 2%) were more frequent in the abnormal group; the majority of these differences failed to show any statistical significance (Table 1).

Median CRP and albumin measured in the normal and abnormal groups were both within normal ranges and the distribution of cancer types was broadly similar (Table 2).

## Discussion

We conducted this pilot study to look for a contributory relationship of vitamin D depletion in relation to chemotherapy toxicity as anecdotally we and others noted poor tolerability among patients with vitamin D depletion, especially, in relation to skin toxicity and mucositis. There is increasing evidence that vitamin D is involved in a number of activities in the body other that its more traditional role in bone modelling and calcium regulation. For example, vitamin D receptor is believed to contribute to the regulation of insulin signalling, the response of macrophages to antigens as well as control of cell proliferation (Dixon *et al*, 2005; Chen *et al*, 2007; Sigmundsdottir *et al*, 2007; Bikle, 2010).

This study was planned to look at a causal relationship between vitamin D levels and chemotherapy toxicities. The long-term aim was to conduct a larger prospective study looking at significance of the same and addressing the correlation of vitamin D replacement therapy to improved chemotherapy toxicity. It was, however, limited by its small sample size, particularly in the normal vitamin D group and was also subject to a degree of bias as vitamin D was predominantly only measured in patients whose levels were expected to be low. Although tumour types were broadly similar across the two groups, the fact that because of this many of the individuals will have been on different chemotherapy regimens can also be viewed as a limiting factor in the interpretation of the data. This is also the case when considering that disease stage was not consistent throughout.

The abnormal group had a number of toxicities observed more often on a numerical basis than the normal group, including thrombosis, hypertension, sensory neuropathy and motor neuropathy, but they were not of statistical significance. On further analysis, by comparing normal and deficient groups, no significant data was found, further reinforcing the view that there is little association between vitamin D level and toxicity encountered during chemotherapy.

We were unable to find any difference between patients with normal and abnormal vitamin D levels. However, interpretation of the data was limited by small patient numbers, especially in the normal group. We believe that bias was minimised by blinding the researchers to patient names and the objective analysis of the assessments but we were unable to determine the vitamin D supplementation status for all patients during their chemotherapy treatment. Despite these limitations the observation that there were no major differences between the two groups suggests, at least in this study, that vitamin D measurement in unlikely to change significantly the management of oncology patients on chemotherapy.

## Figures and Tables

**Table 1:** Side effects for normal and abnormal groups

**Side effects**	**Normal,** ***N*** **(%)**	**Abnormal,** ***N*** **(%)**	***P*** **-value**
Allergic reaction	0 (0)	1 (1)	0.65
Anorexia	2 (5)	13 (7)	0.70
Carpal tunnel syndrome	0 (0)	1 (1)	0.65
Constipation	4 (10)	19 (10)	0.96
Cough	0 (0)	6 (3)	0.23
Desquamation	0 (0)	6 (3)	0.26
Diarrhoea	4 (10)	19 (10)	0.96
Dry skin	7 (17)	19 (10)	0.15
Fatigue	9 (22)	38 (19)	0.67
Fever	0 (0)	1 (1)	0.65
Hand–foot reaction	6 (14)	9 (5)	0.01*
Hearing loss	3 (7)	20 (10)	0.96
Hot flushes	0 (0)	1 (1)	0.65
Hypertension	0 (0)	11 (6)	0.11
Injection site reaction	1 (2)	0 (0)	0.03*
Insomnia	5 (12)	8 (4)	0.03*
Memory loss	0 (0)	1 (1)	0.65
Mood changes	0 (0)	8 (4)	0.19
Motor neuropathy	1 (2)	9 (5)	0.55
Mucositis	4 (10)	11 (6)	0.30
Nail changes	1 (2)	0 (0)	0.03*
Pain	5 (12)	43 (21)	0.18
Painful rash	2 (5)	11 (6)	0.87
Sensory neuropathy	1 (2)	13 (7)	0.31
Taste changes	1 (2)	8 (4)	0.03*
Thrombosis	1 (2)	16 (8)	0.21
Vomiting	0 (0)	12 (6)	0.11
Weight gain	1 (2)	0 (0)	0.03*

* Statistically significant.

**Table 2:** Distribution of cancers in normal and abnormal groups

**Cancer type**	**Normal,** ***N*** **(%)**	**Abnormal,** ***N*** **(%)**
Breast	17 (41)	69 (35)
Colorectal	10 (24)	58 (29)
Lung^a^	5 (12)	19 (10)
Lymphoma/haematological	1 (2)	3 (2)
Head and neck^b^	0 (0)	3 (2)
Gynaecological	0 (0)	11 (6)
Pancreatic and small intestine	3 (7)	15 (8)
Urological^c^	3 (7)	11 (6)
Cancer of unknown primary	1 (2)	4 (2)

a Including mesothelioma.

b Including pyriform sinus and tonsillar fossa.

c Including kidneys, bladder, prostate and testes.
