# Supplementary material for: The relationship between vitamin D and chemotherapy-induced toxicity – a pilot study
Source: Br J Cancer. 2012 May 15;107(1):158–60. doi: 10.1038/bjc.2012.194 (PMC3389405; doi:10.1038/bjc.2012.194)
Supplement: Supplementary Table S1 [file bjc2012194x1.doc]

**Supplementary online material.**

**Table S1**. Toxicity incidence in three vitamin D subgroups.

|  | ***Normal***  ***N (%)*** | ***Insufficient***  ***N (%)*** | ***Deficient***  ***N (%)*** | ***P Value*** |
| --- | --- | --- | --- | --- |
| **Number of patients** | 41 (17) | 97 (40) | 103 (43) |  |
| **Number of patients with grade two toxicity or greater** | 29 (71) | 60 (62) | 76 (74) | 0.24 |
